# The Effect of two Shading Techniques on Value of Zirconia-Based Crowns

**Published:** 2015-06

**Authors:** Ahmad Hassan Ahangari, Kianoosh Torabi Ardakani, Farideh Mahdavi, Mahshid Torabi Ardakani

**Affiliations:** 1 Dept. of Prosthodontics, School of Dentistry, Shiraz University of Medical Sciences, Shiraz, Iran;; 2 Postgraduate Student in Prosthodontics, Dept. of Prosthodontics, School of Dentistry, Shiraz University of Medical Sciences, Shiraz, Iran;; 3 Private Dentist, Shiraz, Iran;

**Keywords:** Zirconia, Color, Crown, Value, Spectrophotometer

## Abstract

**Statement of the Problem:**

By introducing the coloring liquids, it is claimed that it is possible to make the color of frameworks fabricated from zirconium oxide extremely close to the natural tooth color.

**Purpose:**

The purpose of this research was to evaluate the effect of two staining techniques on value changing in zirconia crowns.

**Materials and Method:**

Three groups A, B, and C, each containing ten zirconia crowns, were used. The zirconium cores samples were fabricated by a CAD/CAM device. Group A was left uncolored, Groups B was submerged for two minutes in A2 coloring liquid and Group C was stained with brush. Then all cores were sintered and the porcelain was applied by using the layering technique. Ultimately, the crowns color was determined using a spectrophotometer. Their color changing (∆E) and value changing (∆L) in relation to A2 color were also assessed. The data were analyzed with one-sample t-test, post-hoc Tukey, and one-way ANOVA tests with significant level set at 0.05.

**Results:**

The mean value in all groups was higher than the value obtained from A2 color samples (*p*= 0.001). The highest mean value was 78.31±1.22 belonging to group C (staining with brush) and the lowest mean value was 76.99±0.65 belonging to group B (submerging). The results of post-hoc Tukey regarding both ∆E and ∆L variables showed a significant difference between groups A (uncolored) and C (staining with brush) with P_∆E_=0.006 and P_∆L_=0.039, respectively. A significant difference between group B (submerging technique) and C (staining with brush) were shown when these two variables were compared (P_∆E_=0.001, P_∆L_=0.015).

**Conclusion:**

Due to the higher value increase in surface staining (brush), it is recommended to use the submerging technique for staining zirconia cores.

## Introduction

In recent years, esthetic dentistry has boomed to a great extent as patients’ demands have increased greatly and developments in approaches and new materials have improved clinicians abilities in providing esthetic treatments.[1]

The color compatibility has always been the first and foremost step in restorative and prosthetic dentistry.[2]

Color is a phenomenon resultant of visual perception which depends on the light reflected from or passed through an object.[3]

Visual color matching through making comparison between a patient’s tooth and the standard sample is the most common approach used in clinical dentistry.[4]

Visual evaluation is generally accepted to be unreliable and to have variable outcomes, while instrumental measurements have concrete and quantitative results.[5]

Selecting restorative materials compatible with the natural tooth color has always been one of the most complex issues in restorative dentistry.

Porcelain shade selection guides entirely differ from the restorations that are used in restoring these teeth. People, as well, have different abilities in selecting colors. In addition, changes in the lighting conditions of environment can cause changes in color perception. Besides, porcelain thickness can produce a remarkable effect on the final color.[6]

In order to achieve maximum esthetics, it is essential for the restoration to restore the translucency as well as the color. Translucency of restorative material produces a natural and real appearance in the restorations. Natural teeth demonstrate variable colors and translucencies all over their structures. Therefore, translucency has the same importance as color in the process of color adjustment in achieving restoration esthetics.[7]

Although a majority of restorations are still fabricated from PFM, metal-free restorations have become much more popular.[8] Unique chemical stability, excellent mechanical properties, beautiful color and simultaneous application of CAD/CAM technology have resulted in using zirconia as the selected core material in diverse prosthetic treatments.[9] Zirconia is not completely similar to the tooth color in appearance because it is opaque. To reach the natural appearance of the porcelain and veneer directions and forces, it is possible to color zirconia frameworks. Currently, some factories try to improve the esthetic qualities of this ceramic by pre-colored zirconia cores.[10]

Using liners has been suggested to compensate for the negative effect of the white color of zirconia on the restoration final color. It has been claimed that these liners decrease the strength of zirconia-veneer bond. As a substitute for these liners, newer zirconia coloring liquids which are used before sintering or pre-colored zirconia are recommended, although the advantages of these materials are under discussion.[8]

There is an increasing tendency to use all-ceramic restorations in clinical practice for the excellent esthetics that these restorations provide. Nevertheless, due to the natural white color of zirconium oxide, zirconia blocks are of high value and this issue has caused concerns regarding color compatibility between the color of the restoration and that of the natural tooth, particularly in esthetic areas. However, introducing the coloring liquids has asserted that it is feasible to make the color of zirconium oxide frameworks much more compatible with the natural tooth color. Due to the particular importance of esthetics in modern clinical dentistry and high expectations of patients regarding this issue, and also limited published scientific research on zirconia coloring; more comprehensive researches are needed in this field. Vichi *et al.*[8] has done a review of literature on the color of ceramic and zirconia restorations, and concluded that using zirconia frameworks cannot improve the esthetics of restoration significantly. The white color of zirconia and its low translucency result in limitations in the simulation of the natural tooth appearance and it demands application of feldspathic ceramic layers. According to this research, there is little information regarding the esthetic efficiency of layers on zirconia frameworks. In recent years, zirconia cores staining have received attention from researchers and thus some studies have been done in this regard. Alikhasi *et al.*[10] analyzed the staining effects of the three colors (B3, A3 and D3) on the two axial flexural strength of a type of ceramic with zirconia base (Cercon) and concluded that the staining type can be significantly influential on the flexural strength of this type of ceramic. The present study aimed to analyze the influence of two zirconium cores staining techniques on value change in all-ceramic crowns.

## Materials and Method

This *in vitro* experimental study was performed on the three A, B and C groups of zirconia crowns. There were 10 zirconia crowns in each group. In order to prepare the samples, the right maxillary central incisor tooth on dentiform was prepared to make full coverage crowns. Tooth preparation was performed by a high-speed turbine and round end shoulder finish line of 1 millimeter width and incisal edge reduction of 1.5 millimeters. Then the impression was taken from prepared tooth and its adjacent teeth using additional silicon impression material and a pre-fabricated tray. The impression was poured with high strength dental stone and then a removable die was prepared. In the next phase, 30 zirconia cores were prepared with the thickness of 0.6 mm in the labial surface to 1mm in the lingual surface by using CAD/CAM device (Schutz Dental GmbH; Rosbach, Germany) and zirconia blocks (Vita In-ceram YZ-14; Vivadent, Germany). Frameworks in Group A remained uncolored. In fact, this group was considered as the control group for the evaluation of the effects of coloring liquids on the final color changes of zirconia crowns. Due to the manufacturer’s instructions on the coloring liquid (VITA In-ceram YZ coloring liquid), the frameworks in group B were submerged in A2 coloring liquid for 2 minutes and their moisture was absorbed by a tissue afterwards.

In group C, instead of submerging the crowns, A2 coloring liquid was applied on the outer surface of the zirconia framework by using a brush and then like group B, the moisture of the crowns was absorbed. After the coloring phase, all crowns were sintered and then the A2 color porcelain was applied on the frames in all three groups, using the layering method. In order to make the porcelain thickness even in all crowns, a full wax up was performed. Subsequently, an index was prepared with silicon putty which was a little bigger than the final size of the crown to compensate for the shrinkage produced by porcelain baking. Then, this full wax-up was cut-back to the size needed for the enamel porcelain and a silicon index was taken from that. For applying the porcelain (Vita VM9; Vivadent, Germany), dentin porcelain powder was put in the index taken from the cut-back wax-up and fitted on the frame. Thereafter, the porcelain was baked and the enamel porcelain was put in a full wax-up index, fitted on the dentin porcelain, and then baked. Then the crowns were glazed. Eventually, in each group the color of the crowns was determined by a spectrophotometer device (Dentsply DeguDent GmbH; Germany) and its ∆E (color changing) and ∆L (value changing) in relation to A2 color in vita classic shade guide were evaluated. The statistical methods used to compare the mean color changing and mean value changing among the tested groups included post-hoc Tukey, one- way ANOVA and one-sample t-test. One- way ANOVA analysis was used for the comparison of mean color changing (∆E) and mean value changing (∆L) among the three groups which were under study. One- sample t-test was used for comparing mean value of each group with the reference quantity of 73.70 (vita classic A2 color sample value). 

## Results

The results demonstrated a significant difference between at least two groups from the three groups under study. The results of post-hoc Tukey test, regarding both ∆E and ∆L variables, indicated a significant difference between the group A (without staining) and group C (staining with brush) with P_∆E_=0.006 and P_∆L_=0.039, respectively. A statistically significant difference was also observed between the two groups B (submerging technique) and C (staining with brush) when these two variables were compared (P_∆E_=0.001, P_∆L_=0.015).

No statistically significant difference was observed between groups A (without staining) and B (submerging technique) (Table 1).

**Table 1:** Comparing color changing (∆E) and value changing (∆L) in different groups

**∆E**	** ∆L**	
**Group**	**Mean ± SD**	**Mean ± SD**
A	3.47 ±1.00^*^	3.74 ± 0.84^*^
B	3.29 ± 0.65^*^	3.50 ± 0.59^*^
C	4.61 ± 1.22^**^	5.01 ± 1.01^**^

*no significant difference, ** significant difference,

A= uncolored, B= submerging technique, C= staining with brush The mean difference is significant at 0.05 level

The results of one-sample t-test demonstrated a significant difference between the mean value in all three groups and the A2 color sample value (*p*= 0.001). The mean value in all three groups was higher than the A2 color sample value. The highest mean value was 78.31±1.22 belonging to group C (staining with brush) and the lowest mean value was 76.99 ±0.65 belonging to group B (submerging).

Group C (staining with brush) showed the most difference from the A2 color sample value, while group B (submerging) had the least difference.

According to the results of this test, there was a statistically significant difference between group B (submerging) mean value and C (staining with brush). However, the mean value in neither of these two groups showed a significant difference from group A (Table 2).

**Table 2:** One- sample t-test results for comparing mean values with reference quantity

**Group**	**Mean ± SD**	**P value**
A	77.17±1.00	<0.001
B	76.99±.65	<0.001
C	78.31±1.22	<0.001

A= uncolored, B= submerging technique, C= staining with brush The mean difference is significant at 0.05 level

## Discussion

The white color of zirconia is an obstacle in reaching the desired esthetics and thus its surface has to be covered.[8] Currently, limited information is available on staining the zirconia frameworks.[11-12] In their research, Alikhasi *et al.*[10] analyzed the influence of the surface staining technique on the flexural strength of Cercon zirconia. In addition, Hjerppe *et al.*[13] used the submerging technique for staining zirconia frameworks and analyzed its effect on the biaxial strength and surface microhardness. Nevertheless, no study was available regarding the liners and coloring liquids effects on color and value changes in all ceramic crowns. The present study made a comparison between the effects of the two techniques, submerging and staining with brush (surface staining), on color value changing of zirconia crowns.

To match the color of restoration or prosthesis with natural dentition, two intricate procedures of color selection and color reproduction should be followed.[8] Visual estimation of color mismatch should be minimized to a great extent and the color selection procedure should be improved by using shade- taking devices. Principally, there are two different categories of electronic instruments which can be used to measure the general color; colorimeters and spectrophotometers.[14] In the present study, a spectrophotometer was employed to determine the sample color.

In general, the Commission International de l’Eclairage (CIE) defined color space is commonly employed for measuring the instrumental color. CIE L value which measures the lightness of any given object can be calculated on a scale of zero to 100. Based on this scale, the CIE L value is zero for a perfect black and 100 for a perfect reflecting diffuser. The color difference between each pair of specimens can be calculated to determine the amount of color match, using the following equation: ∆E = [(∆l)^2^ + (∆a)^2^ + (∆b)^2^]^1.2^.[15]

The results of this research demonstrated that the value increased in all A, B, and C groups which was a predictable outcome. Porcelain shade selection guides completely differ from the restorations which are applied in restoring these teeth. Normally, porcelain shade- selection guides are in the shape of tabs, which have a high thickness (4mm). Most shade selection guides do not have metal or zirconia basis and are fabricated from ceramic materials and differ from the final restoration,[6] while the frameworks in all three groups under study were from zirconia. As it is known, one of the problems with zirconia is its opacity which explains the higher value in all three groups compared to the A2 color sample in vita-shade classic guide.[6] Moreover, according to the results of the present study, group C (staining with brush) had the most and group B (submerging) had the least color changing (∆E) and value changing (∆L) from the A2 color sample. The authors contend that the submerging technique which was applied in group B resulted in the penetration of the coloring liquid into all parts of the crown and consequently lead to the diffused reflection of the light and a decrease in the value, whereas in group C, the color was applied exclusively to the cores outer surface with a brush which caused an increase in the surface reflection (mirror reflection) of the light and the value, as well.

Johnston *et al.*[8] in 1989, evaluated color difference acceptability threshold of ∆E = 3.7. In the present study, ∆E was 3.74 in group A (uncolored), 3.50 in group B (submerging technique) and 5.01 in group C (staining with brush). Regarding the results of Johnston *et al.*,[8] only group B was in an acceptable range according to clinical factors. This finding was in line with other results of the present study and consistent with other studies that recommended the submerging technique strongly.[8]

The material will be more opaque if the majority of light passing through the ceramic spreads out extremely and be reflected diffusely. In contrast, when there is only part of the light which is dispersed and most of it is transmitted diffusely, the material is more translucent.[16]This issue, also, confirms the result of the present study.

According to Aboushelib *et al.,*[8] coating natural zirconia framework with veneer ceramic helps duplicate the required color more accurately. In case of colored zirconia, liner material or deep chroma dentin should be applied to reproduce an appropriate color. In addition, they claimed that application of pre-colored zirconia frameworks was not superior to the use of standard natural zirconia.

The number of scientific reports on the color of zirconia core restorations is very limited; therefore, this subject needs to be investigated extensively in future studies. 

## Conclusion

The results of the present research demonstrated that the type of zirconia cores staining technique has an influence on all ceramic crowns value. Therefore, it is recommended to consider staining technique as one of the influential factors on the final color of zirconia crowns.

It is recommended to use the submerging technique of staining Zirconia cores in order to reproduce closer value of natural teeth. 
